# Quantum Hash function and its application to privacy amplification in quantum key distribution, pseudo-random number generation and image encryption

**DOI:** 10.1038/srep19788

**Published:** 2016-01-29

**Authors:** Yu-Guang Yang, Peng Xu, Rui Yang, Yi-Hua Zhou, Wei-Min Shi

**Affiliations:** 1 College of Computer Science and Technology, Beijing University of Technology, Beijing 100124, China; 2 State Key Laboratory of Information Security (Institute of Information Engineering, Chinese Academy of Sciences, Beijing 100093, China; 3 Beijing Key Laboratory of Trusted Computing, Beijing 100124, China; 4 National Engineering Laboratory for Critical Technologies of Information Security Classified Protection, Beijing 100124, China

## Abstract

Quantum information and quantum computation have achieved a huge success during the last years. In this paper, we investigate the capability of quantum Hash function, which can be constructed by subtly modifying quantum walks, a famous quantum computation model. It is found that quantum Hash function can act as a hash function for the privacy amplification process of quantum key distribution systems with higher security. As a byproduct, quantum Hash function can also be used for pseudo-random number generation due to its inherent chaotic dynamics. Further we discuss the application of quantum Hash function to image encryption and propose a novel image encryption algorithm. Numerical simulations and performance comparisons show that quantum Hash function is eligible for privacy amplification in quantum key distribution, pseudo-random number generation and image encryption in terms of various hash tests and randomness tests. It extends the scope of application of quantum computation and quantum information.

With the rapid development of quantum communication, quantum key distribution (QKD) is the most mature branch of quantum communication. The goal of QKD is to create an absolutely secure key between two communicating parties. QKD generally contains three steps: (1) raw key sifting, (2) error reconciliation, and (3) privacy amplification. As an important step of QKD, the privacy amplification process is implemented by adopting universal hash functions^1^. However, these hash functions are generally constructed based on mathematics complexity and thus they are computationally secure. Because the fundamental principles of quantum mechanics ensure lots of quantum cryptographic protocols^2^,^3^,^4^ with unconditional security, this stimulates us to consider the privacy amplification problem in the context of quantum information, and intend to get a more secure solution to the privacy amplification process.

In this paper, we construct a quantum Hash function (QHF) by subtly modifying the quantum walks (QW) model^5^,^6^,^7^,^8^,^9^,^10^,^11^,^12^,^13^ and it can be used for the privacy amplification process of QKD systems with higher security by means of the physical principles of quantum mechanics. As a byproduct, QHF can also be used for pseudo-random number generation due to its inherent chaotic dynamics and further we propose a novel QHF-based image encryption algorithm. Numerical simulations and performance comparisons show that QHF is eligible as a hash function for privacy amplification in QKD, pseudo-random number generation and image encryption in terms of various hash tests and randomness tests.

Compared to the QW-based algorithm^14^, the novelty of the present QHF-based scheme lies in that the constructed QHF can be not only used for pseudo-random number generation and further image encryption, but also used for the privacy amplification process of QKD systems with higher security. It extends the scope of application of quantum computation and quantum information.

## Results

### The construction of QHF

QHF can be constructed by subtly modifying QW. QW has two models: discrete QW and continuous QW^5^. The basic discrete QW includes two quantum systems: walker and coin. The state of the walker-coin system is denoted by a vector in the Hilbert space 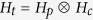, where the subscripts *p* and *c* stand for the walker and the coin, respectively. The motion of the walk is conditioned by the coin state via a conditional shift operator


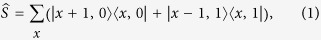


where the summation symbol denotes the sum over all possible positions. The evolution of the total quantum system can be implemented by repeating the global unitary operator


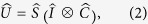


where 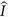 is the identity operator and 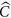 is the coin operator applied on the coin state. Hence the final state 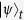 after *t* steps is expressed by


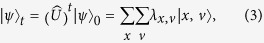


and the probability of locating the walker at position *x* after *t* steps is


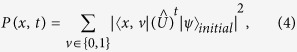


where 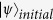 is the initial state of the total quantum system.

In a discrete-time QW, the coin operator is fixed. The resulting probability distribution relies on only the initial coin state and the step number. Suppose the coin operator at each step depends on a binary string, i.e., *message*, and accordingly a QHF is constructed, similar to that in Ref.^15^. The input of the constructed QHF is a binary string, i.e., *message* and the resulting probability distribution is used as the output hash value. The coin state is the control parameter so the constructed QHF is a keyed one. The *n*th bit of the *message* controls the *n*th step of the walk. Here we introduce two coin operators, i.e., the Grover operator 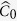
16 and the coin operator 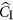
17 in equation (5) and equation (6) respectively. The *message* bit “0” denotes 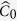 and “1” for 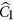.


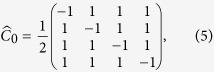



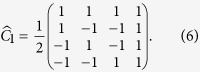


The construction process of the QHF is as follows:

1. Select the parameters (*n*, (*α*, *β*, *χ*, *δ*)) and the *message* with arbitrary length.
2. Run the one-dimensional two-particle discrete-time QW on a circle under the control of the *message* and generate the output hash value, i.e., the probability distribution. Here *α*, *β*, *χ*, *δ* are the amplitudes of the initial coin state 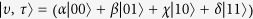. *n* is the node number of a circle.
3. Multiply all values in the resulting probability distribution by 10^8^ modulo 256 to form a binary string as a secret key *K*, i.e., the hash value.

### The hash property of the proposed QHF

In this section, we performed several hash tests to evaluate the performance of the proposed QHF. The *message* can be randomly chosen as shown in Supplemental materials.

### Statistical distribution of hash value

Based on the principles of quantum mechanics, the security of the QHF can be proved partly by the uniform distribution of the hash value. The plots of the ASCII codes of the *message* and its hash value are shown in Supplementary Fig. S1 online. Supplementary Fig. S1(a) demonstrates that the ASCII code of the *message* is located within a small range, but in Supplementary Fig. S1(b), the hash value of the *message* in hexadecimal format is scattered uniformly.

### Sensitivity of hash value to message

C1, C2, C3 and C4 represent the *message*, and the *message* with tiny modifications respectively. The results listed below show the high sensitivity to the *message* and the tiny changes.

Condition 1: The original *message*;

Condition 2: Change the 8^th^ bit from 0 to 1;

Condition 3: Delete the last bit of the *message*;

Condition 4: Insert a bit in front of the 100^th^ bit.

The corresponding 128-bit hash value in the hexadecimal format is given by:

Condition 1: 8AE72983687E9AD1B6FCA54546AE7799;

Condition 2: 3BD58DB7B86827AE6323E6E496A634A8;

Condition 3: E9678E1EA9180A8EE01AA008EB46E989;

Condition 4: E9277B78B3D62DEB77839DB9E90F210D.

The plots of the hash values are shown respectively in Supplementary Fig. S2 online and it is clearly indicated that any tiny modification to the *message* or the key will cause a substantial change in the final hash value.

### Statistical analysis of diffusion and confusion

The diffusion and confusion tests are performed as follows:

1. Select a *message* and generate the corresponding hash value.
2. Change one bit of the *message* randomly and generate a new hash value.
3. Compare the two hash values and count the changed bits called *B*_*i*_.
4. Repeat steps (1) to (3) *N* times.

The corresponding distribution and the histogram of *B*_*i*_are shown respectively in Supplementary Figs S3(a) and S3(b) online, where *N* = 10,000.

Minimum changed bit number 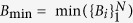;

Maximum changed bit number 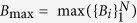;

Mean changed bit number 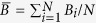;

Mean changed probability 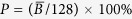;

Standard variance of the changed bit number 
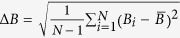
;

Standard variance of the changed probability 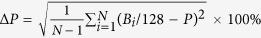.

Next the diffusion and confusion tests are performed with *N* = 1024, 2048, 10,000, respectively, as shown in Table 1. We concluded from the tests that the mean changed bit number *B* and the mean changed probability *P* are close to the ideal value 64 and 50% respectively. Δ*B* and Δ*P* are very little, so that it demonstrates the stability of diffusion and confusion. The excellent statistical effect ensures that it is impossible to forge plaintext-cipher pairs given known plaintext-cipher pairs.

### Collision analysis

It is hard to provide a mathematical proof on the capability of collision resistance of chaotic hash functions. Thus, we performed the following test for collision resistance:

1. Select an original message randomly and generate the corresponding hash value in ASCII format.
2. Choose a bit in the message randomly and change its value.
3. Generate a new hash value.
4. Compare these two hash values and count the number of ASCII characters with the same value at the same location.

**Figure 1:**
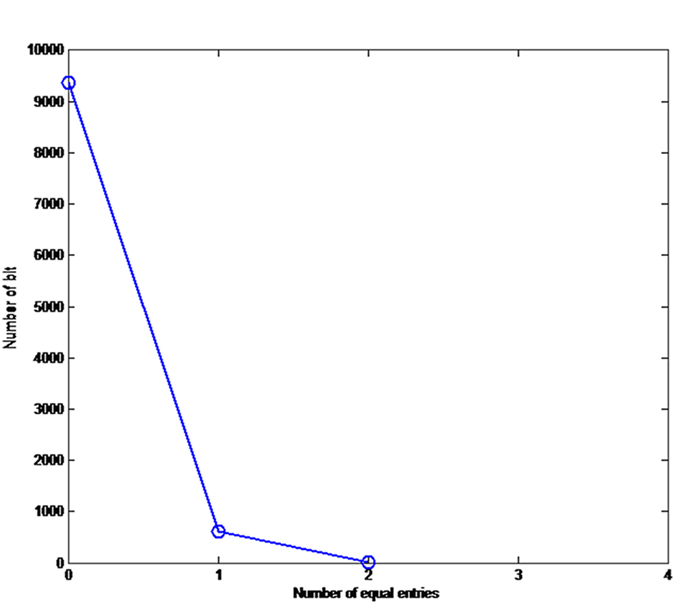
Distribution of the number of positions where the ASCII characters are identical in the 128-bit hash values generated for test, where *N* = 10,000. (see text in the section entitled Results).

Moreover, the absolute difference of the two hash values, i.e., *d*, and the theoretical number of *ω* with different values through *N* independent tests, i.e., *W*_*N*_(*ω*) can be computed according to the following formulas:


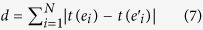



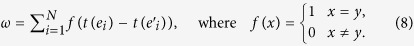



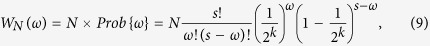


where *e*_*i*_ and *e’*_*i*_ are the *i*th entries of the original and new hash values, respectively. The function *t*(·) converts the entries to their equivalent decimal value. We run this test *N* = 10, 000 times, and listed the maximum, minimum, and mean of *d* in Supplementary Table 1 online respectively. In equation (8), *ω* = 0, 1, …, s. The experimental values of *W*_*N*_(*ω*) in the proposed scheme are: *W*_*N*_(0) = 9367, *W*_*N*_(1) = 617, *W*_*N*_(2) = 16, and *W*_*N*_(*ω*) = 0 for *ω* = 3, 4, …, 16 respectively. The distribution of the number of ASCII characters with the same value at the same location in the hash value is displayed in Fig. 1.

### Uniform distribution on hash space

In order to check the distribution capacity in hash space, similar to that in ref. 18, we generated two hash values according to the method described in previous subsection and then counted the number of the changed bits at each location. The minimum, maximum and mean of changed bit numbers are 4072, 5689 and 4973.5, respectively for *N* = 10,000. The statistical results for *N* = 10,000 are shown in Supplementary Fig. S4 online. The mean of the changed bit number 4973.5 is very close to the ideal value 5000, which accounts for half of the test times. It can be concluded that the hash value is distributed uniformly in the hash space as all the changed bit numbers are around the ideal value. Obviously, this demonstrates the resistance against statistical attack.

### Resistance to birthday attack

Similar to collision resistance, the birthday attack is mainly aimed to find two messages with identical hash values with less than 2^*n*/2^ trials (*n* is the size of hash value). According to the current computing power, the size of hash value should be greater than 128 to ensure the birthday attack complexity is greater than 264. In our proposed hash scheme, the length of hash value is 128-bit and it can be easily extended to 256-bit or 512-bit. So the attack difficulty is at least 2^64^, which is huge enough to resist brute force attack and birthday attack. Also in the proposed algorithm, in order to analyze the security, several tests including the SP800-22 test and the collision tests were applied. Therefore, the results of the tests, the size of the hash value, and the collision resistance of the proposed algorithm suggest that the birthday attack is almost impossible and that the proposed algorithm is resistant against this type of attack.

### Speed analysis

Our proposed hash algorithm does not need to pad bits. The time required to generate a hash value is closely related to the length of the message. The algorithm is simulated in MATLAB on a PC with Intel(R) Core(TM) i3-2370M CPU 2.40 GHz 2 GB RAM running on Windows 7 professional OS. The average speed of our algorithm is 0.2 Mbit/s. Although the speed of the proposed algorithm is lower than the traditional hash functions such as SHA-1 and MD5^19^, it is acceptable for practical use. At the same time, the algorithm is so flexible since the length of hash value can be 128, 256, or 512 bits. Moreover, because of the excellent properties of quantum parallel computing, the speed of the proposed algorithm will increase exponentially in the quantum computing environment. For example, finding the prime factorization of an *n*-bit integer is thought to require 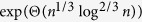 operations using the best classical algorithm. In contrast, a quantum algorithm can accomplish the same task using 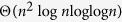 operations^20^. That is, a quantum computer can factor a number exponentially faster than the best known classical algorithms.

### Flexibility

Our hash scheme is based on the QW model. Although the proposed hash function is constructed as a keyed one, we can also regard it as an unkeyed one if the initial parameters of the coin state of QHF act as the constants. Besides we can also get a 256-bit or 512-bit hash function with a slight change in the original version, similar to refs 21,22.

### Resistance to meet-in-the-middle attack

The meet-in-the-middle attack is valid for the block cipher encryption mode. Block cipher mode allows the use of a cipher key for encrypting more than one block of data. In contrast, in our proposed algorithm, we use just the nonlinear quantum map to construct the hash function, so the attack is useless for the proposed algorithm.

### Resistance to forgery attack

Most of the parallel hash function algorithms have a mixing section in their structure which usually uses the XOR operation for preventing forgery attack. Unfortunately, some of these algorithms are broken by such an attack^23^,^24^. In the proposed algorithm, the state evolution of the total quantum system can be implemented by repeating the sequence of the coin flipping operator and the conditional shift operator step by step according to the message(so-called discrete time). That is, the *n*th bit of the *message* controls the *n*th step of the walk, i.e., applying the Grover operator 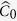
16 or the coin operator 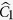
17 on the coin state. This leads to high complexity in mixture and can resist forgery attack in any section of the proposed algorithm.

### Security analyses of the QHF-based pseudo-random number generator (PRNG)

QHF can also be used for pseudo-random number generation due to its inherent chaotic dynamics. To analyze the pseudo-randomness of the QHF-based PRNG, we analyzed its statistical properties and some quantifiers were proposed. The quantifiers are mainly classified into two classes: (i) quantifiers based on information theory^25^,^26^,^27^, (ii) quantifiers based on recurrence plots^28^,^29^.

### Statistical complexity measure

**Figure 2:**
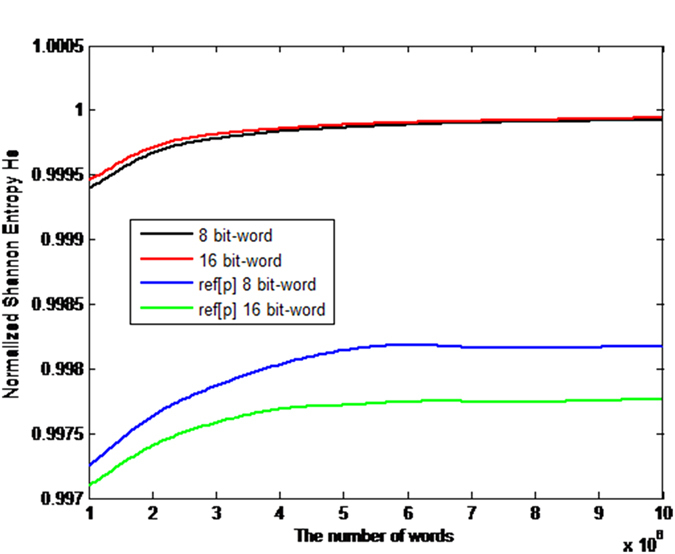
Comparisons in terms of Normalized Shannon entropy *H*_*S*_. The black and red curves denote the Normalized Shannon entropy *H*_*S*_ for the number of 8 bits and 16 bits-words respectively in our proposal, while the blue and green curves represent the Normalized Shannon entropy of the QW-based scheme^14^ for the number of 8 bits and 16 bits-words respectively. (see text in the section entitled Results).

**Figure 3:**
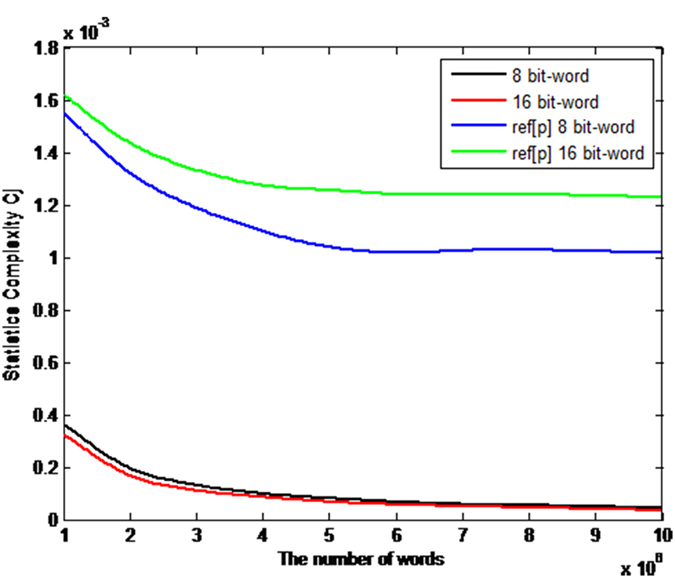
Comparisons in terms of intensive statistical complexity measure *C*_*J*_ respectively. The black and red curves denote the intensive statistical complexity measure *C*_*J*_ for the number of 8 bits and 16 bits-words respectively in our proposal, while the blue and green curves represent the intensive statistical complexity measure *C*_*J*_ of the QW-based scheme^14^ for the number of 8 bits and 16 bits-words respectively. (see text in the section entitled Results).

Complexity is a measure of off-equilibrium ‘order’. Statistical complexity measures (SCM) were proposed as quantifiers of the degree of physical structure in a signal^25^,^30^,^31^. Based on the method of Ref. 32, we analyzed the statistical complexity of the QHF-based PRNG. The intensive SCM (*C*_*J*_[*P*]) can be considered as a quantity that characterizes the probability distribution *P* associated with the time series generated by the dynamical system^32^. It quantifies not only randomness but also the presence of correlational structures^31^,^32^. The measure of statistical complexity *C*_*J*_[*P*] is defined as^32^:


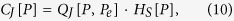


where the normalized entropic measure 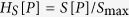 is associated with the probability distribution *P*, with 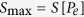 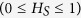 for the equilibrium distribution *P*_*e*_ and *S* is the Shannon entropy. The disequilibrium *Q*_*J*_ is defined in terms of the Jensen-Shannon divergence^26^,^32^ by


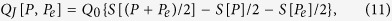


with *Q*_0_ being the normalization constant 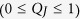. Thus, the disequilibrium *Q*_*J*_ is an intensive quantity. Following the methodology proposed by Bandt and Pompe^33^, the comparisons between our proposal and the QW-based PRNG^14^ in terms of the normalized entropy *H*_*S*_ and the intensive statistical complexity *C*_*J*_ as functions of the number of 8 bits and 16 bits-words are shown in Figs 2 and 3 respectively. When the number of words of the analyzed sequence increases, the statistical complexity and the normalized entropy tend to 0 and 1 respectively. It is shown that given the same words, our scheme has better statistical complexity and normalized entropy than the QW-based PRNG^14^.

### Recurrence plots

Recurrence is a fundamental property of dynamical systems, which can be exploited to characterize the system’s behavior in phase space. In 1987, Eckmann *et al.* introduced a powerful tool for visualization and analysis of recurrences called recurrence plot (*RP*)^28^. *RP* is a two-dimensional representation in which both axes are time ones. The recurrence of a state appearing at two given times *t*_*i*_, *t*_*j*_ is pictured in the two-dimensional graph by means of a black dot.

To visualize the recurrences of states of a dynamical system, the *RP* of a trajectory 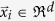 can be formally expressed by the matrix


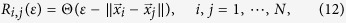


where *N* is the number of measured points 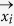, *ε* is a threshold distance, 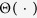 is the Heaviside function (i.e. 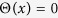, if *x* < 0, and 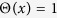 otherwise) and 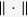 is a norm.

*RP*s for various values of the *message* exhibit visually the recurrences of the QHF-based PRNG (Supplementary Fig. S5 online). We used an embedding dimension *m* = 2 and the delay τ = 1. The threshold distance *ε* is set to be 10% of the mean phase space radius σ. It is shown that the QHF-based PRNG with different *message*s causes a rather homogeneous *RP* with numerous single points and some short, diagonal or vertical lines.

Because the visual impact produced by the *RP* is insufficient to demonstrate the quality of the QHF-based PRNG because of the ‘small-scale’ structures^29^, several measures of complexity which quantify the small scale structures in *RP*s, have been proposed^34^,^35^,^36^ and are known as recurrence quantification analysis (*RQA*). In this paper, these measures based on the recurrence point density and the diagonal and vertical line structures are considered.

### Measures based on the recurrence density

The simplest measure of the *RQA* is the recurrence rate (*RR*)


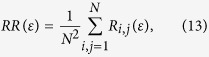


which is a measure of the density of recurrence points in the *RP*. Furthermore, in the limit *N* → ∞, RR is the probability that a state recurs to its *ε*-neighbourhood in phase space. For PRNGs, the ideal value would be *RR* = 0. But in practice, in order that the quantifier may make sense, a larger *ε* should be adopted to avoid the situation in which no points are found in the recurrence plot. It is shown that the value of the *RR* ranges from 0.00595 to 0.00662 for different *messages* (see Supplementary Fig. S6 online). It exhibits the good randomness of the QHF-based PRNG.

### Measures based on diagonal lines

The measures are related to the histogram 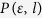 of the diagonal line lengths *l*, given by


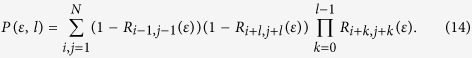


Supplementary Fig. S7 online shows the histogram of the diagonal line lengths of the *RP* in Supplementary Fig. S5 online with the parameter *message* = 50. It is shown that the diagonal line lengths are mainly very short exhibiting the good randomness.

Processes with uncorrelated or weakly correlated behaviors cause none or very short diagonals, whereas deterministic processes cause longer diagonals and less single, isolated recurrence points. Therefore, the ratio of recurrence points that form diagonal structures (of at least length *l*_min_) to all recurrence points


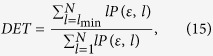


is introduced as a measure for determinism (or predictability) of the system. The threshold *l*_min_ excludes the diagonal lines which are formed by the tangential motion of the phase space trajectory.

A diagonal line of length *l* means that a segment of the trajectory is rather close during *l* time steps to another segment of the trajectory at a different time; thus these lines are related to the divergence of the trajectory segments. The average diagonal line length


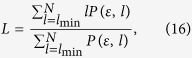


is the average time that two segments of the trajectory are close to each other, and can be interpreted as the mean prediction time.

Another *RQA* measure considers the length *L*_max_ of the longest diagonal line found in the *RP*,


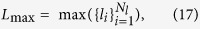


where 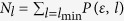 is the total number of diagonal lines. These measures are related to the exponential divergence of the phase space trajectory. The faster the trajectory segments diverge, the shorter the diagonal lines are.

The measure entropy refers to the Shannon entropy of the probability 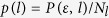 to find a diagonal line of exactly length *l* in the *RP*, where 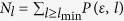 is the total number of diagonal lines.


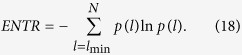


*ENTR* reflects the complexity of the *RP* in respect of the diagonal lines, e.g. for uncorrelated noise the value of *ENTR* is rather small, indicating its low complexity, as shown in Supplementary Fig. S8 online.

### Measures based on vertical lines

The total number of the vertical lines of the length *v* in the *RP* is then given by the histogram


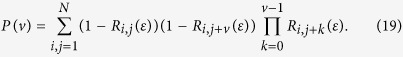


Supplementary Fig. S9 online shows the histogram of vertical line lengths of the *RP* in Supplementary Fig. S5 with the parameter *message* = 50. It is shown that the vertical line lengths are mainly very short exhibiting the good randomness.

Analogous to the definition of the determinism in equation (15), the ratio between the recurrence points forming the vertical structures and the entire set of recurrence points can be computed,


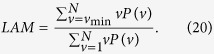


The computation of *LAM* is realized for those *v* that exceed a minimal length *v*_min_ in order to decrease the influence of the tangential motion. *LAM* will decrease if the *RP* consists of more single recurrence points than vertical structures.

The average length of vertical structures is given by


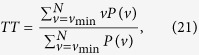


and is called trapping time. *TT* estimates the mean time that the system will abide at a specific state or how long the state will be trapped.

Finally, the maximal length of the vertical lines in the *RP*


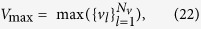


can be defined, analogously to the standard measure *L*_max_ (*N*_*v*_ is the absolute number of vertical lines).

**Figure 4:**
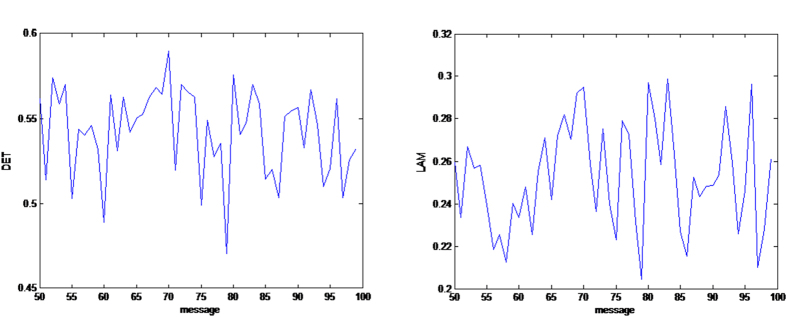
Selected *RQA* measures *DET*, *L*_max_. *DET* and *LAM* change with different *message*s are shown in (**a**,**b**) respectively. (see text in the section entitled Results).

**Figure 5:**
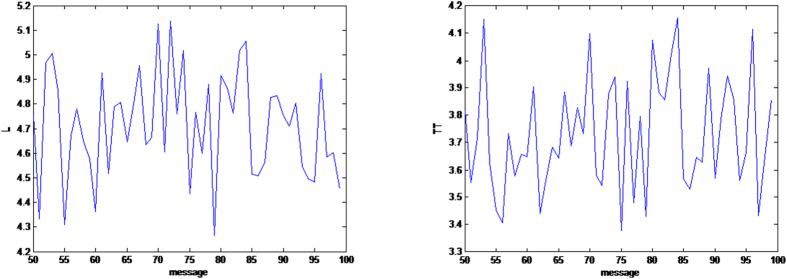
Selected *RQA* measures *L* and *TT*. *L* and *TT* change with different *message*s are shown in (**a**,**b**) respectively. (see text in the section entitled Results).

**Figure 6:**
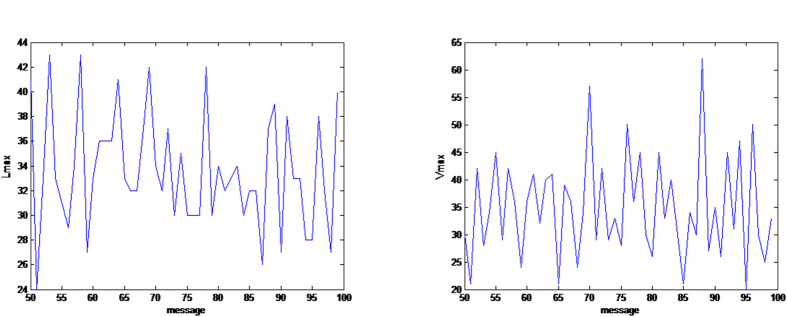
Selected *RQA* measures *LAM* and *V*_max_. *L*_max_ and *V*_max_ change with different *message*s are shown in (**a**,**b**) respectively. (see text in the section entitled Results).

Figures 4, 5, 6 give some selected *RQA* measures for different values of the *message* and demonstrates the good statistical properties of the QHF-based PRNG.

### Degree of non-periodicity

In order to study the non-periodicity in the QHF-based PRNG, the scale index analysis (SIA) is carried out which is introduced by Benìtez *et al.*^37^. The SIA method is often used as a framework to enhance the general performance of cryptosystems in designing new chaos-based cryptosystems and PRNGs. For example, recently Akhshani *et al.* proposed a new scheme for generating good PRNGs based on quantum logistic map^38^. They used the SIA technique to assess the degree of non-periodicity of the chaotic sequences of the quantum map. The SIA technique is based on the continuous wavelet transform (CWT) and the wavelet multi-resolution analysis^39^. To study the non-periodicity of the QHF-based PRNG^40^, we assumed that the key sequence *f* generated by QHF is compactly supported and is defined over a finite time interval *T* = [*a*, *b*]. The CWT of *f* at time *u* and scale *s* is defined as^39^:


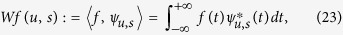


and it provides the frequency component (or details) of *f* corresponding to the scale *s* and time location *t*. Supplementary Fig. S10 online shows the time frequency decomposition of *f* in the time-frequency plane which provided by the wavelet transform given in equation (23).

The wavelet transform given in equation (23), provides a time frequency decomposition of *f* in the time-frequency plane.

The scalogram of *f* is defined as follows:


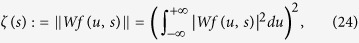


where 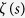 is the energy of the CWT of *f* at scale *s*. The scalogram is a useful tool for studying a signal, since it allows the detection of its most representative scales or frequencies^37^,^40^. The innerscalogram of *f* at a scale *s* can be defined by:


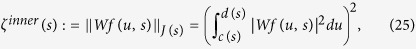


where 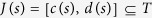 is the maximal subinterval in *T* for which the support of 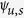 is included in *T* for all 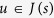. As the length of *J*(*s*) depends on the scale *s*, the values of the inner scalogram at different scales cannot be compared. Therefore, the inner scalogram should be normalized as follows^37^:


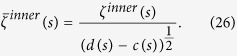


Supplementary Fig. S11 online shows that the normalized inner scalogram can be a valuable tool for detecting the non-periodicity of the signals, where a signal with details at every scale is non-periodic.

The scale index of *f* in the scale interval [*s*_0_, *s*_1_] can be defined by:


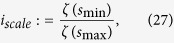


where *s*_max_ is the smallest scale such that 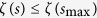 for all 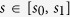, and *s*_min_ the smallest scale such that 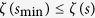 for all 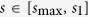. The scale index will be zero or close to zero for periodic sequences and close to one for highly non-periodic sequences^37^.

**Figure 7:**
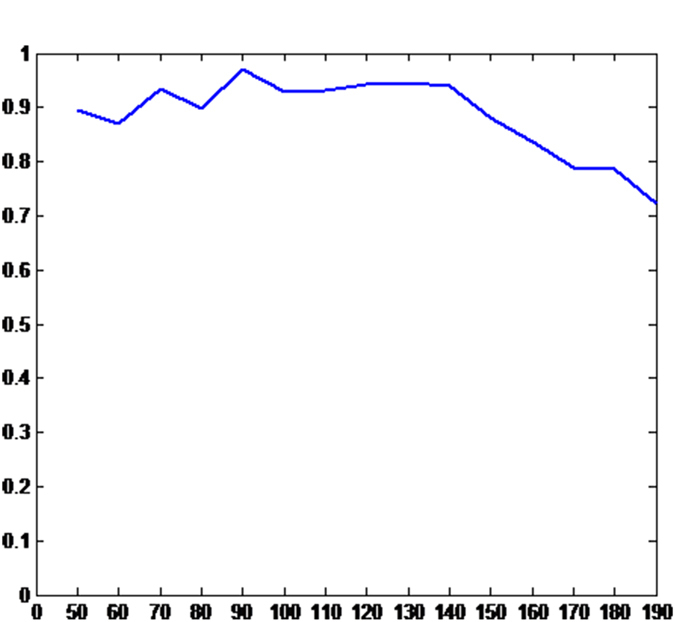
The scale index of the QHF-based key sequence for different *messages*. It can be concluded that, the value of the scale index is larger than 0.7 and the best value of the scale index is up to 0.98. Thus, the key sequence in this state is highly non-periodic and it can be used for any PRNG purposes. (see text in the section entitled Results).

In Fig. 7, the SIA of the QHF-based key sequence is presented. It can be concluded that the best value of the scale index is up to 0.98. Thus, the key sequence in this state is highly non-periodic and it can be used for any PRNG purposes.

### The application of the QHF-based PRNG to image encryption

The proposed image encryption algorithm consists of the following major steps:

#### Step 1: Initialization step

In this step we choose the parameters (*n*, (*α*, *β*, *χ*, *δ*)) and the *message*, then generate a random sequence *S* by running our proposed PRNG given by


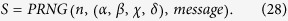


Each value of the sequence ***S*** is a floating-point number ranging from 0 to 1.

#### Step 2: Encryption procedure

1. Divide the original image *I* with size *M* × *N* into four parts with the same size and determine the encryption order of four image blocks according to the size of the first four values of the sequence *S*. For example, 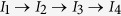 if 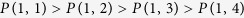. Here we take Buddha image as an example (see Supplementary Fig. S12 online).
2. The encryption process is a two round one (Supplementary Fig. S13 online) i.e.,

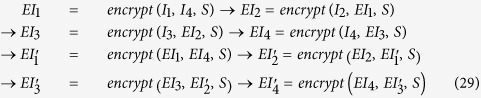


Then we describe the encrypt function 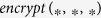 by taking *I*_2_ as an example.

1. Sum the gray values of *EI*_1_ to produce a binary sequence *V*, use *V* as the *message* to generate a new random sequence *S*_1_ as follows.


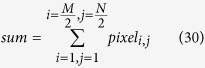


where *pixel*_*i*,*j*_ represents the gray value of the pixel in the *i*^th^ row and *j*^th^ column of *EI*_1_.


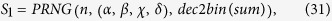


where *dec*2*bin* is a function to convert the decimal to binary sequence.

2. Do the tensor product of *S* and *S*_1_ to obtain a new sequence and transform it into a two-dimensional matrix of size 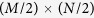.


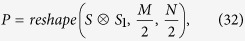


where 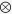 is tensor product operator.

3. Multiply all values in the *P* matrix by 10^8^ modulo 256 to form a new matrix *P*′.


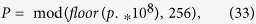


4. Make *P*′ exclusive-OR (XOR) with *I*_2_ and get


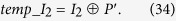


5. Sort the elements in *P*′ in ascending order, and obtain the index vector.


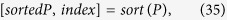


where *sortedP* is the sorted matrix and index is the index vector, i.e., 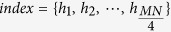.

6. Shuffle the elements in *temp*_*I*_2_ and get encrypted image block *EI*_2_ for the first round.


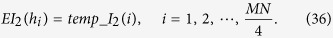


At last we can get the final encrypted image by executing the encrypt function as the order shown in *Step 2*.

#### Step 3: Decryption procedure

In the decryption process, we use the secret key to determine the decryption order, shown as follows.


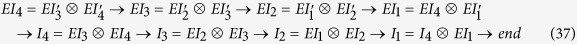


Here, 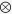 represents the decryption operation and it is the reverse of the encryption process.

### Experimental simulations

Experiments are performed on a laptop with Intel(R) Core(TM) i3-2370M CPU 2.40 GHz 2 GB RAM running on Windows 7 professional equipped with the MATLAB R2012a environment and the Python 2.7.8. To test our encryption method for security and robustness, we choose a group of images with size 512 × 512, which were taken by Yu-Guang Yang and list them and their corresponding encrypted images and decrypted images in Supplementary Figs S12–S18.

### Key space analysis

A desirable encryption scheme should have a sufficiently large key space to resist brute-force attacks. Here the encryption key can be represented by (*n*, (*α*, *β*, *χ*, *δ*), *message*), where *n* denotes the number of nodes in a circle. *α*, *β*, *χ*, *δ* are complex numbers and also control parameters of the coin state satisfying the normalization constraint: 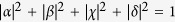. *Message* is a binary string as an input of the proposed QHF, which can have an infinite length theoretically. Although there is an infinite key space theoretically, because of finite precision of digital computers, the key space actually turns out to be finite. Considering that the calculation precision is 10^−14^, the size of key space for initial conditions and control parameters would be roughly 2^325^, which is large enough for any encryption purposes and is also large enough to resist all kinds of brute-force attacks.

### Histogram analysis

Histogram is a very important security measure for evaluating the security of an image encryption algorithm. The histograms of seven images and the corresponding cipher images are shown in Supplementary Figs S12–S18 online. It is shown that the histograms of the cipher images are nearly uniform and significantly different from the ones of the original images. Therefore they provide no clue for attackers in a statistical analysis attack.

### Correlation analysis

A desirable encryption scheme should generate the cipher image with rather low correlation between adjacent pixels. By randomly selecting 10000 pairs (in horizontal, vertical and diagonal directions respectively) of adjacent pixels from the original image and the cipher image, respectively, we test the correlation between adjacent pixels, and draw the correlation distribution of adjacent pixels in the Buddha image and its cipher image in Supplementary Fig. S21 online, respectively. It is shown that the original image has strong correlation, but the cipher image is quite random. The encryption scheme improves the security of the test images greatly.

We also calculate the correlation coefficient *r*_*xy*_ of adjacent pixels of the original image and the cipher image given by


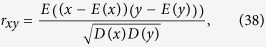


where *E*(*x*) and *D*(*x*) are the expectation and variance of variable *x*, respectively.

We compute the correlation coefficients of the seven images and their cipher images. From Supplementary Table S2 online, we can see that the average correlation coefficients of the encrypted images are 0.0010, 0.0012, 0.0023 and they are very close to zero. The result demonstrates that our scheme is effective.

### Comparison with other image encryption techniques

Experimental results of the proposed image encryption scheme were compared with six typical image encryption techniques, i.e., chaos-based image encryption^41^, optics-based image encryption^42^, hash-based image encryption^43^, quantum image encryption^44^, the QW-based image encryption^14^, and the image encryption based on quantum logistic map^45^, respectively. From Supplementary Tables S3–S8 online, we can see that the images encrypted by our algorithm have lower correlation compared with the six image encryption schemes^14^,^41^,^42^,^43^,^44^,^45^. In addition, we also compared our algorithm with two recently published schemes^46^,^47^ and see Supplementary Tables S9 and S10 online for details.

### Differential attack analysis

In general, two common performance measures are used to test the influence of a little change in the original image on the cipher image, i.e., the *number of pixels change rate* (*NPCR*) and the *unified average changing intensity* (*UACI*). *NPCR* is expressed by


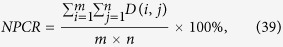


where


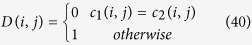


and *c*_1_ and *c*_2_ are two cipher images with size *m* × *n*.

*UACI* is defined by


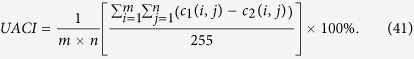


In our tests, we just put the gray value of grid (100,100) minus one in the seven images in Supplementary Figs S12–S18 with size 512 × 512, respectively. From Supplementary Table S11 online, we can see that *NPCR* is 99.61% and *UACI* is 33.46% in our algorithm which implies that it is highly sensitive to the original image and is robust against differential attacks. In addition, we also compared our proposal with other image encryption schemes^41^,^43^,^45^,^46^,^47^ and see Supplementary Tables S12–S17 online for details. From these tables, it is indicated that the proposed algorithm can resist the differential attack. The secret key in our algorithm is related to the image self. So it makes a good performance in this field.

### Key sensitivity analysis

First, we encrypted Buddha image with the key *n* = 1, [*α*, *β*, *χ*, *δ*] = [0, 0, 0, 1], message = [1, 0, 1, 0, …, 1, 0, 1, 0…], and obtained the cipher image in Supplementary Fig. S22(a). Then we encrypted Buddha image by making a little change of the message and got the cipher image in Supplementary Fig. S22(b). We also draw the differential image of Supplementary Fig. S22(a) and S22(b) in Supplementary Fig. S22(c). By calculation, we got that the difference (*NPCR*) between Supplementary Fig. S22(a) and Supplementary Fig. S22(b) is 99.59%(see Supplementary Tables S18 online), which implies the encryption process is quite sensitive to the encryption key.

Second, we encrypted Buddha image by the key *n* = 1, [*α*, *β*, *χ*, *δ*] = [0, 0, 0, 1], *message* = [1, 0, 0, 1, …, 1, 0, 1, 1…], and then decrypted the resulting cipher image with the correct key (see Supplementary Fig. S22(d)) and the wrong key with a little change of the message = [1, 0, 0, 1, …, 1, 1, 1, 1…] (see Supplementary Fig. S22(e)), respectively. We calculated out the difference (*NPCR*) between Supplementary Fig. S22(d) and Fig. S22(e) is 99.61% (see Supplementary Tables S18 online). Therefore, the decryption process is also highly sensitive to the decryption key.

In the same way, we also calculated the NPCR of other test images from Supplementary Figs S12–S18, and listed the results in Supplementary Table S18 online. It is found that the results are approximately 99.60%, which shows that the proposed algorithm is of good key sensitivity to images.

### Information entropy analysis

The information entropy is often used to measure the randomness of the cipher images. The entropy *H*(*x*) of a message source *m* is given by


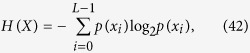


where *p*(*x*_*i*_) represents the probability of the occurrence of symbol *x*_*i*_.

From Supplementary Table S19 online, we can see the average information entropy of our cipher images is very close to the theoretical value 8. We also compared our algorithm with the algorithm based on quantum logistic map^45^ in terms of information entropy(Supplementary Table S20 online). This implies that the information leakage in the encryption process is negligible and the proposed algorithm is stable and secure against information entropy attack.

### Randomness test analysis

Next, we used NIST SP800-22 and TestU01^48^ tools to test the randomness of the cipher images. Each test produces a *P-value* in [0, 1]. If the *P-value* is higher than a preset threshold *α*, it means that the cipher image passes the test. In our tests, we set *α* = 0.01 and Buddha’s cipher image with size 1024 × 1024. *α* = 0.01 implies that the cipher image can be inferred to be random with 99% probability if it passes the test. From Supplementary Table S21 online, we can judge that our proposed algorithm passes the NIST SP800-22 tests.

However, when we applied the most stringent test by TestU01^48^ on the cipher images, surprisingly, the TestU01 test cannot be done successfully. Maybe the coin flipping operators were not chosen properly. Therefore, the future work will focus on the improvement of our proposal.

### Speed performance analysis

Speed is an important factor for evaluating the performance of an image encryption algorithm. For the proposed image encryption algorithm, we measured the time cost in the operating environment: Windows 7, Matlab R2012a, Intel(R) Core(TM) i3-2370M CPU 2.40 GHz 4 GB RAM. In our algorithm, the time is mainly spent on shuffling pixels, we can figure out that the time complexity of our algorithm is 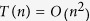 and the average time cost for encrypting images of size 512 × 512 is 0.015 seconds or so. Therefore, our algorithm is fast enough for practical applications.

## Discussion

The main contribution of the work is to construct a hash function used for the privacy amplification process of QKD systems with higher security by means of the physical principles of quantum mechanics. As a byproduct, QHF can also be used for pseudo-random number generation due to its inherent chaotic dynamics and further we propose a novel QHF-based image encryption algorithm.

The QHF is in fact constructed in the quantum context. Because the practical quantum computation device is unavailable, we have to do the simulations by MATLAB software on a classical computer in order to demonstrate the performance of the constructed QHF. However, in fact the properties of quantum parallel computation cannot be simulated precisely on a classical computer. So both randomness and speed of the proposed scheme seem not acceptable for cryptographic purpose. Fortunately, with the rapid development of the field of quantum computation, maybe the practical quantum computer is possible in the future. When the QHF operates on a practical quantum computer, the computation speed will be increased exponentially because of the properties of quantum parallel computation.

## Methods

### The construction of a QHF by modifying the one-dimensional two-particle discrete-time QW on a circle

A one-dimensional two-particle discrete-time QW on a circle describes the QW of two walkers whose motions are restricted to the circle. The operators 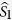 and 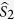 of two-particle QW on circles becomes


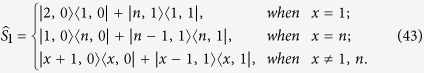


Here 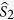 is similar to 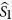. The total conditional sift operator 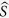 is denoted as 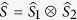.

When the *i*th bit of the message is 0(1), the *i*th step of the walk executes with the interaction 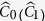. For example, if the message, *m* is ‘0100110’, then the final state evolves


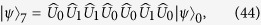


where 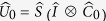 and 
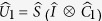
. The initial state of the total quantum system 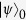 is given by


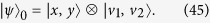


Here


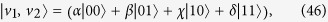


where 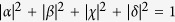.

## Additional Information

**How to cite this article**: Yang, Y.-G. *et al.* Quantum Hash function and its application to privacy amplification in quantum key distribution, pseudo-random number generation and image encryption. *Sci. Rep.* **6**, 19788; doi: 10.1038/srep19788 (2016).

## Supplementary Material

Supplementary Information

## Figures and Tables

**Table 1:** The static number of changed bit *B*_*i*_.

	*N* = 1024	*N* = 2048	*N* = 10,000	Mean
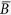	63.5654	63.5864	64.2894	63.8137
*P*(%)	49.6605	49.6769	50.2261	49.8545
Δ*B*	5.4616	5.5841	5.6686	5.6314
Δ*P*	4.3881	4.3626	4.4286	4.3931
*B*_min_	45	44	43	44
*B*_max_	81	83	89	84.3333

The mean changed bit number *B* and the mean changed probability *P* are very close to the ideal value 64 bit and 50% respectively. Δ*B* and Δ*P* are very little, so that it demonstrates the stability of diffusion and confusion. The excellent statistical effect ensures that it is impossible to forge plaintext-cipher text pairs given several known plaintext-cipher text pairs.
