# Supplementary material for: Quantum Hash function and its application to privacy amplification in quantum key distribution, pseudo-random number generation and image encryption
Source: Sci Rep. 2016 Jan 29;6:19788. doi: 10.1038/srep19788 (PMC4731770; doi:10.1038/srep19788)
Supplement: Supplementary Information [file srep19788-s1.pdf]

Supplemental materials

Quantum Hash function and its application to privacy amplification in quantum key distribution, pseudo-random number generation and image encryption

Yu-Guang Yang, Peng Xu, Rui Yang, Yi-Hua Zhou, Wei-Min Shi

The message can be randomly chosen as follows:

“0 0 1 1 0 0 0 0 0 0 1 0 1 1 1 1 1 0 1
0 1 1 0 0 0 1 1 0 1 1 0 1 0 1 0 0 0 0
1 0 1 0 0 0 1 0 0 0 1 0 1 0 1 0 1 0 0
0 1 1 1 0 1 1 1 1 1 0 1 0 0 1 0 0 1 1
0 1 0 1 1 0 1 1 1 1 0 0 0 1 1 0 1 1 1
1 1 1 1 0 0 0 0 0 0 0 1 1 1 1 1 0 0 1
0 1 1 1 0 0 1 1 1 0 0 1 0 0 1 0 0 1 0
1 0 1 1 0 0 0 0 0 0 1 0 1 0 1 0 1 1 0
0 1 1 0 1 0 1 1 0 1 1 1 1 1 0 0 0 1 1
1 0 1 0 0 0 1 0 0 0 0 0 1 1 1 1 1 1 1
1 0 1 1 1 1 0 1 0 0 1 1 1 1 1 0 1 0 1
1 1 1 1 1 1 1 1 0 0 1 0 1 0 1 1 0 1 0
0 0 0 0 0 0 0 0 1 0 1 1 0 1 0 0 0 1 1
0 0 1 1 0 0 1 0 0 0 0 1 1 0 1 1 1 0 1
1 1 1 1 1 0 0 0 0 1 0 0 0 0 1 0 1 1 1
0 0 0 0 1 0 1 0 1 1 1 1 0 0 0 1 1 0 0
0 0 1 0 0 0 1 1 0 1 0 1 1 1 0 1 0 0 0
1 0 0 0 0 0 1 0 0 0 0 0 1 0 0 0 0 0 1
1 1 0 0 1 1 1 0 1 0 0 1 0 0 1 1 1 1 1
1 0 1 1 0 0 1 0 1 1 0 0 1 0 1 1 1 0 0
0 1 1 1 0 1 0 0 1 0 1 1 1 1 0 1 1 1 0
0”

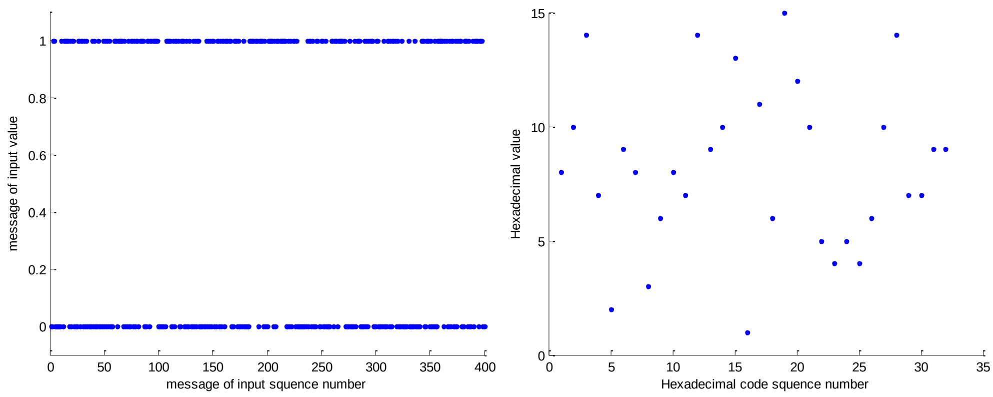

Supplementary Figure S1| (a) Distribution of the original message in ASCII. **(b)** Distribution of the hash value in hexadecimal format.

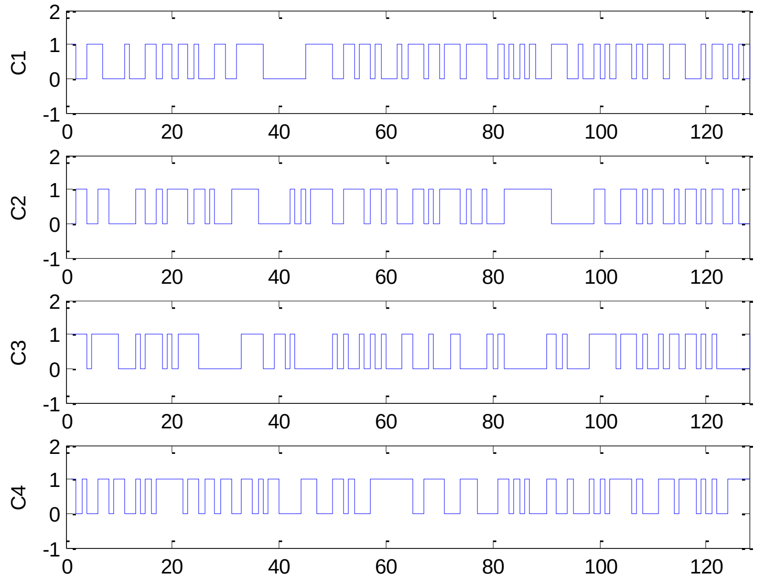

Supplementary Figure S2| Plots of the 128-bit hash value C1, C2, C3 and C4

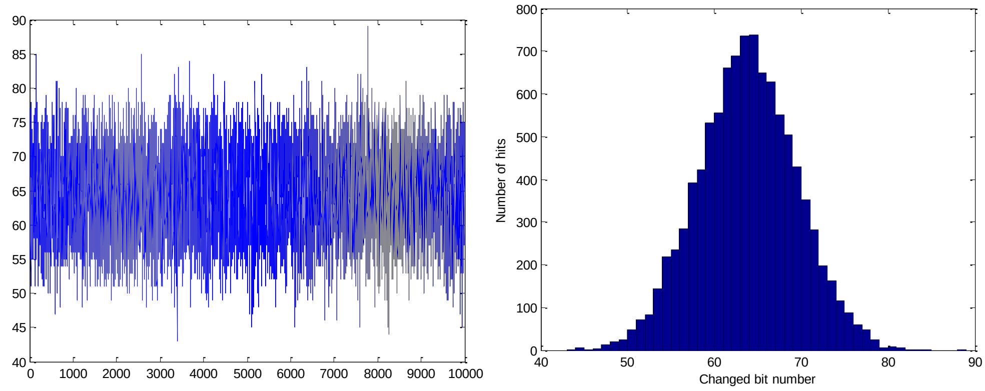

(a) (b)

Supplementary Figure S3| Distribution of the number of the changed bits. (a) plot of B_i. (b) histogram of B_i.

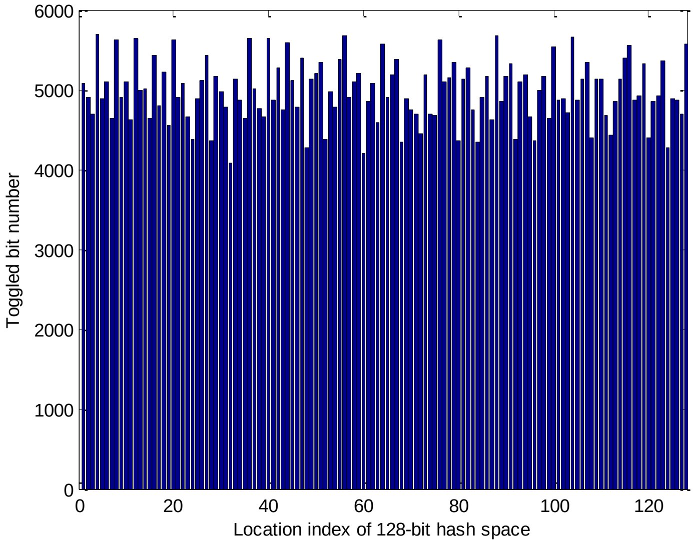

Supplementary Figure S4| Distribution of the hash value in hash space. The mean, maximum and minimum of toggled bit number are 4973.5, 5689 and 4072 respectively for N = 10,000.

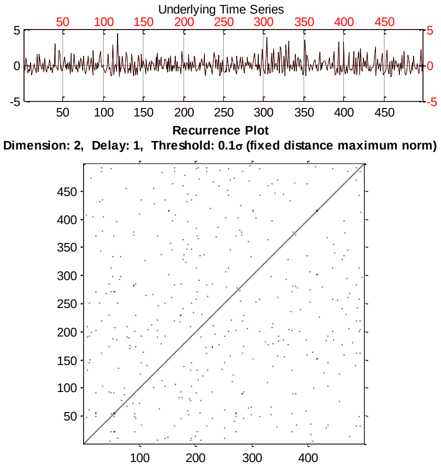

message = 50

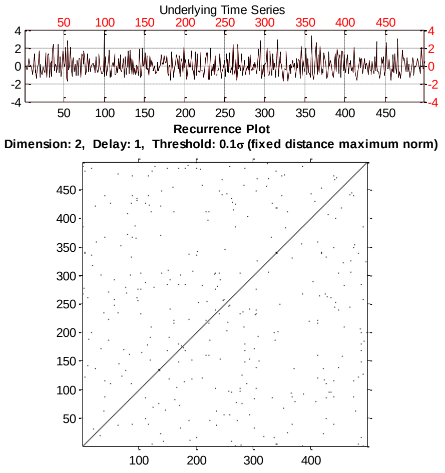

message = 60

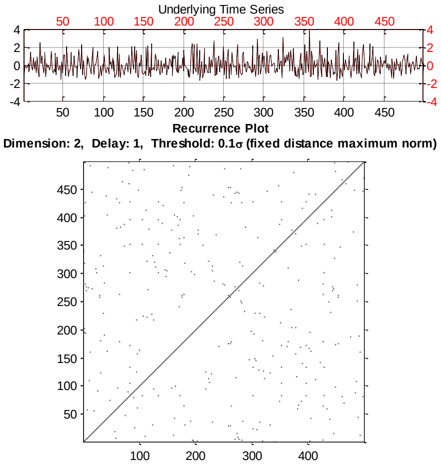

message = 70

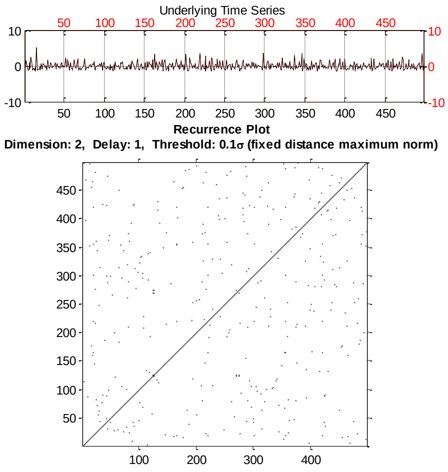

message = 80

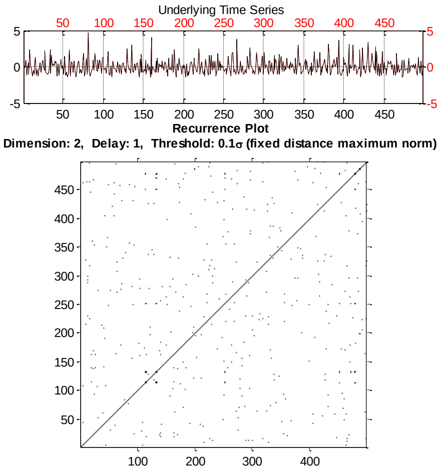

message = 90

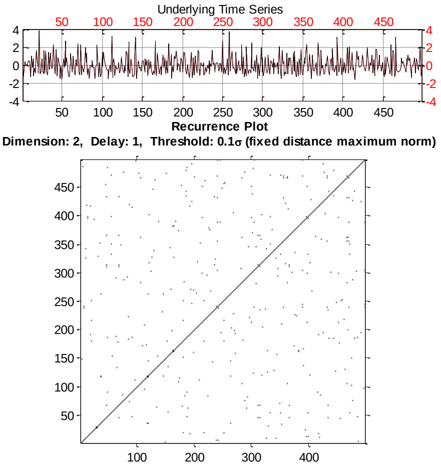

message = 100

Supplementary Figure S5| *RP*s for various values of the control parameters *message*: (a) *message* = 50, (b) *message* = 60, (c) *message* = 70, (d) *message* = 80, (e) *message* = 90, (f) *message* = 100; with the embedding dimension m = 2, time delay τ = 1, threshold ε= 0.1σ, and L_∞ -norm.

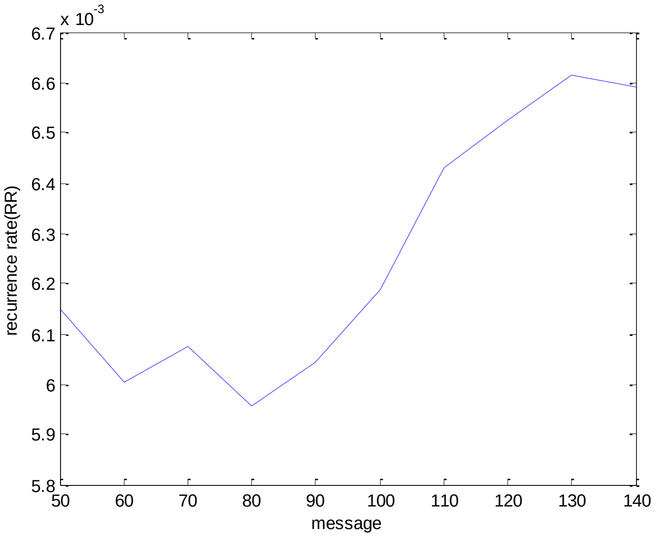

Supplementary Figure S6| RRs with different *messages*.

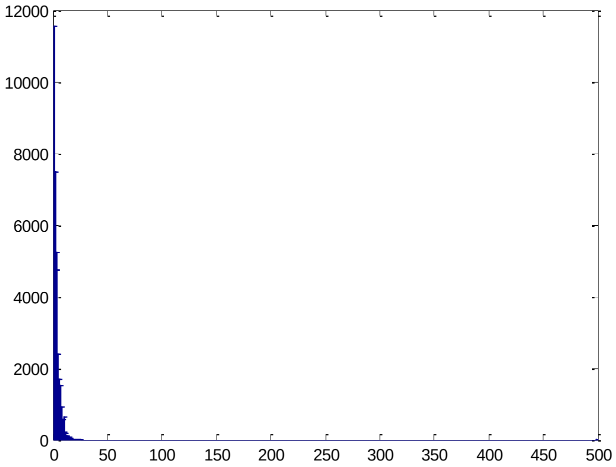

Supplementary Figure S7| The histogram of the diagonal line lengths.

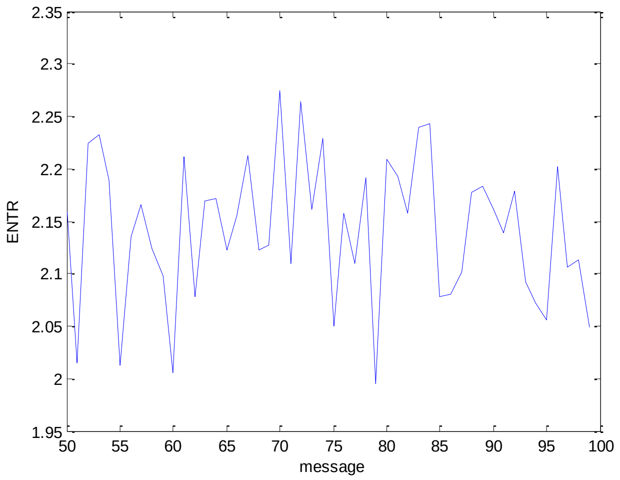

Supplementary Figure S8| The measure entropy *ENTR*.

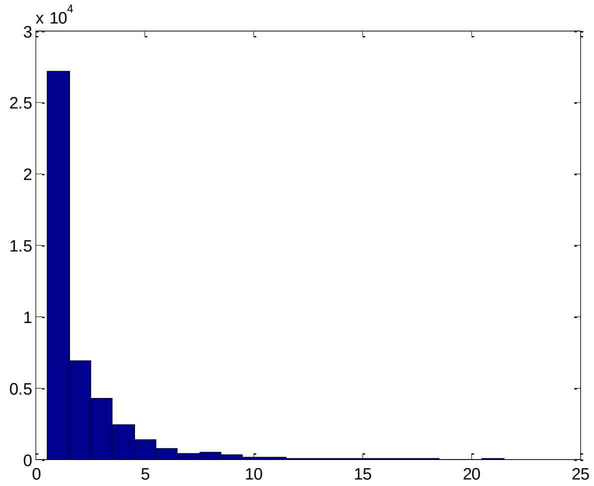

Supplementary Figure S9| The histogram of the vertical line lengths.

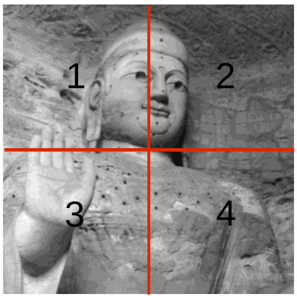

Supplementary Figure S10| the sketch of the original image divided into four parts

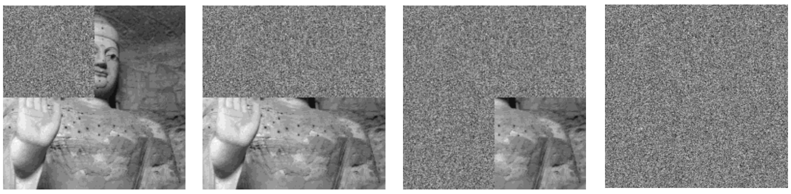

a b c d

Supplementary Figure S11| The first round of the image encryption process. a) Encrypt block 1 with the original image block 4 as the secret key, b) Encrypt block 2 with the encrypted image block 1 as the

secret key, c) Encrypt block 3 with the encrypted image block 2 as the secret key, d) Encrypt block 4 with the encrypted image block 3 as the secret key.

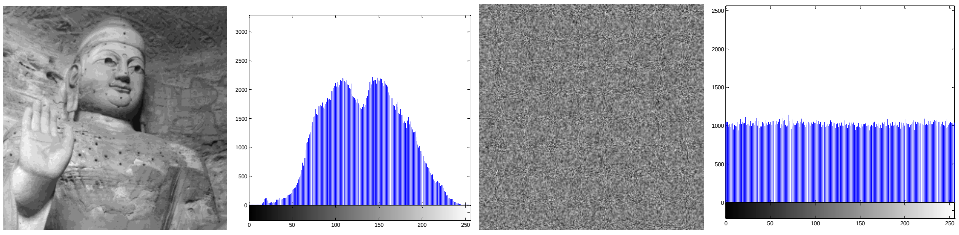

a b c d

Supplementary Figure S12| (a) Buddha image (b) Histograms of Buddha image (c) cipher image (d) Histograms of cipher image. We acknowledge Yu-Guang Yang who took the paragraph in (a).

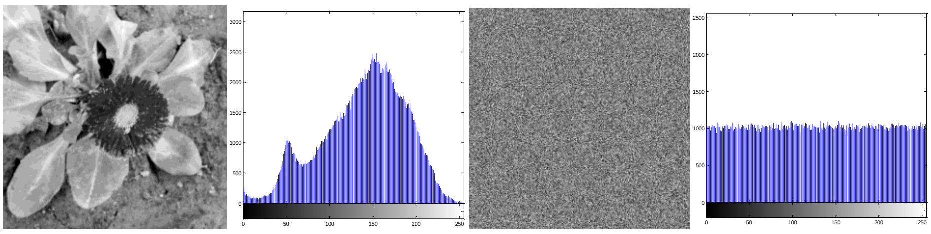

a b c d

Supplementary Figure S13| (a) Flower image (b) Histograms of Flower image (c) cipher image (d) Histograms of cipher image. We acknowledge Yu-Guang Yang who took the paragraph in (a).

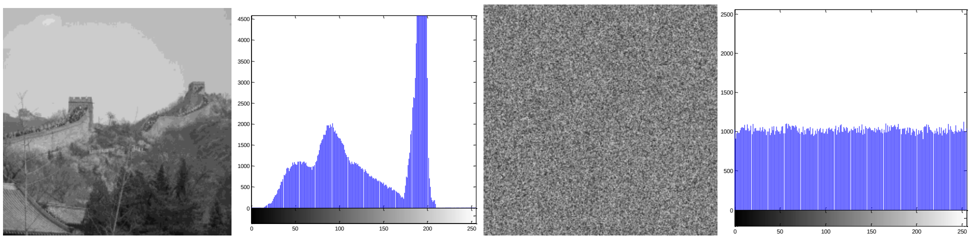

a b c d

Supplementary Figure S14| (a) GreatWall image (b) Histograms of GreatWall image (c) cipher image (d) Histograms of cipher image. We acknowledge Yu-Guang Yang who took the paragraph in (a).

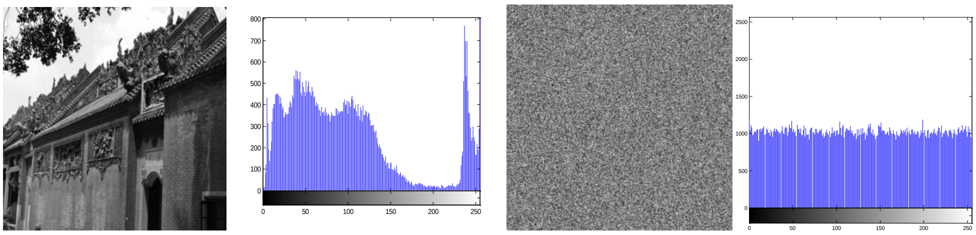

a b c d

Supplementary Figure S15| (a) House image (b) Histograms of House image (c) cipher image (d) Histograms of cipher image. We acknowledge Yu-Guang Yang who took the paragraph in (a).

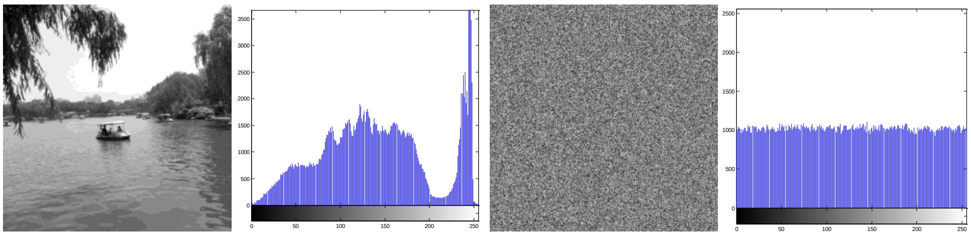

a b c d

Supplementary Figure S16| (a) Lake1 image (b) Histograms of Lake1 image (c) cipher image (d) Histograms of cipher image. We acknowledge Yu-Guang Yang who took the paragraph in (a).

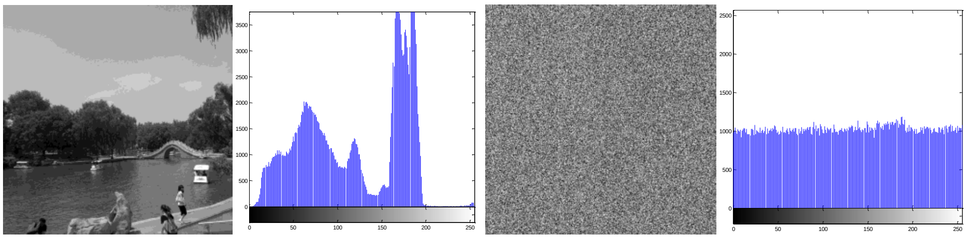

a b c d

Supplementary Figure S17| (a) Lake2 image (b) Histograms of Lake2 image (c) cipher image (d) Histograms of cipher image. We acknowledge Yu-Guang Yang who took the paragraph in (a).

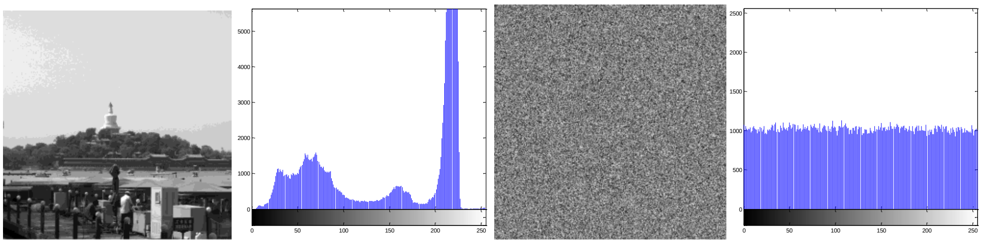

a b c d

Supplementary Figure S18| (a) WhiteTower image (b) Histograms of WhiteTower image (c) cipher image (d) Histograms of cipher image. We acknowledge Yu-Guang Yang who took the paragraph in (a).

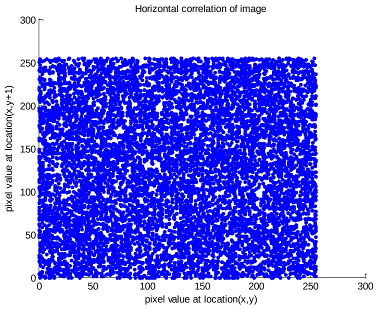

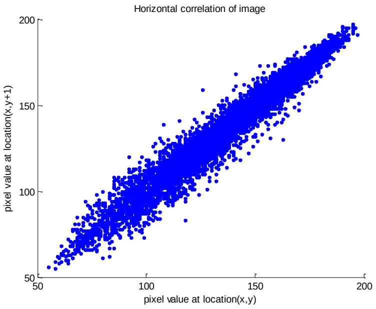

a b

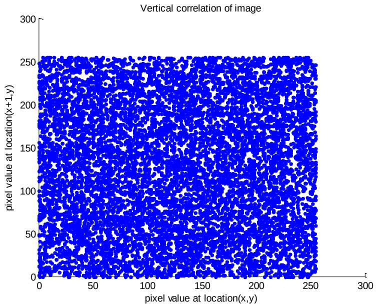

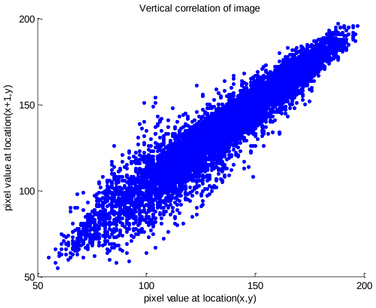

c d

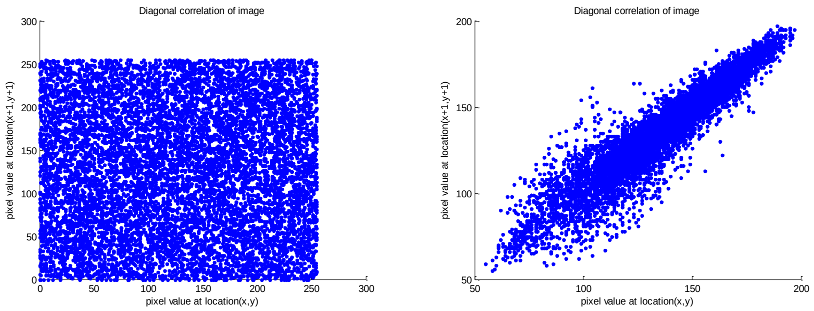

e f

Supplementary Figure S19| Correlation analyses. (a), (c) and (e) show the distribution of the horizontally, vertically and diagonally adjacent pixels in the Buddha image, respectively. (b), (d) and (f) show the distribution of the horizontally, vertically and diagonally adjacent pixels in the encrypted image, respectively.

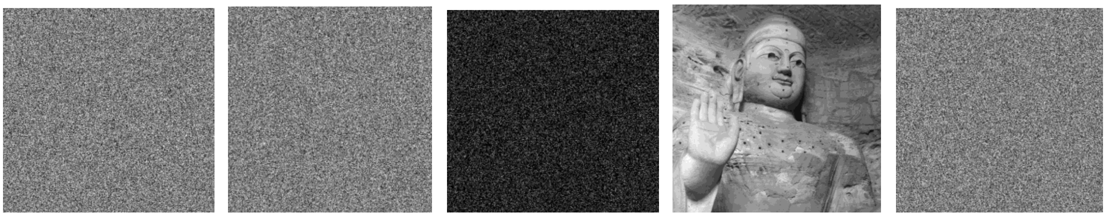

a b c d e

Supplementary Figure S20| Key sensitivity tests. (a) Cipher image. (b) Cipher image with a little difference from encryption key. (c) Differential image between (a) and (b). (d) Decrypted image of (a) with the correct key; (e) Decrypted image of (a) with a little difference from the decryption key. We acknowledge Yu-Guang Yang who took the Buddha image in Supplementary Fig.S12(a) online and all figures in Supplementary Figure S20 online were obtained by simulations using Matlab software by Peng Xu.

Supplementary Table S1| Maximum, minimum and mean of the absolute difference of two hash values.

Absolute difference *d*	Minimum	Maximum	Mean
File	505	2231	1395.3

Supplementary Table S2| Comparison of correlation coefficients of the plain-images and the corresponding cipher-images encrypted by our algorithm

Algorithm / Images	Original image			Our algorithm		
	Horizontal correlation	Vertical correlation	Diagonal correlation	Horizontal correlation	Vertical correlation	Diagonal correlation
Buddha	0.8665	0.7586	0.7261	-0.0004	0.0009	0.0006
Flower	0.9719	0.9850	0.9593	0.0015	-0.00009	-0.00001
GreatWall	-0.9792	-0.9826	0.9680	-0.0016	-0.00008	0.0007
House	0.8597	0.9591	0.8418	-0.0004	0.0007	-0.0035
Lake1	0.9834	0.9903	0.9737	0.0018	0.0020	0.0013

Lake2	0.9890	0.9912	0.9802	0.0015	0.0006	0.0021
WhiteTower	0.9714	0.9745	0.9532	-0.0001	0.0041	0.0053
**Average**	**0.9458**	**0.9160**	**0.9146**	**0.0010**	**0.0012**	**0.0023**

Supplementary Table S3| Comparison of correlation coefficients between our algorithm and the Chaos-based algorithm[41]

Algorithm / Images	Our algorithm			Chaos-based algorithm		
	Horizontal correlation	Vertical correlation	Diagonal correlation	Horizontal correlation	Vertical correlation	Diagonal correlation
Buddha	-0.0004	0.0009	0.0006	-0.0186	-0.0023	0.0041
Flower	0.0015	-0.00009	-0.00001	-0.0078	-0.0009	0.0011
GreatWall	-0.0016	-0.00008	0.0007	-0.0471	-0.0326	0.0659
House	-0.0004	0.0007	-0.0035	-0.0541	-0.0248	0.0569
Lake1	0.0018	0.0020	0.0013	-0.0468	-0.0166	0.0266
Lake2	0.0015	0.0006	0.0021	-0.1271	-0.0906	0.1852
WhiteTower	-0.0001	0.0041	0.0053	-0.1469	-0.0340	0.0682
**Average**	**0.0010**	**0.0012**	**0.0023**	**0.0641**	**0.0288**	**0.0583**

Supplementary Table S4| Comparison of correlation coefficients between our algorithm and the Optics-based algorithm[42]

Algorithm / Images	Our algorithm			Optics-based algorithm		
	Horizontal correlation	Vertical correlation	Diagonal correlation	Horizontal correlation	Vertical correlation	Diagonal correlation
Buddha	-0.0004	0.0009	0.0006	0.0145	0.0290	0.0155
Flower	0.0015	-0.00009	-0.00001	0.0449	0.0707	0.0487
GreatWall	-0.0016	-0.00008	0.0007	0.0285	0.0748	0.0278
House	-0.0004	0.0007	-0.0035	0.0190	0.0550	0.0214
Lake1	0.0018	0.0020	0.0013	0.0362	0.0877	0.0345
Lake2	0.0015	0.0006	0.0021	0.0906	0.1452	0.0918
WhiteTower	-0.0001	0.0041	0.0053	0.0250	0.0673	0.0235
**Average**	**0.0010**	**0.0012**	**0.0023**	**0.0370**	**0.0757**	**0.0376**

Supplementary Table S5| Comparison of correlation coefficients between our algorithm and the hash-based algorithm[43]

Algorithm / Images	Our algorithm			Hash-based algorithm		
	Horizontal correlation	Vertical correlation	Diagonal correlation	Horizontal correlation	Vertical correlation	Diagonal correlation
Buddha	-0.0004	0.0009	0.0006	0.0109	0.0221	0.0044

Flower	0.0015	-0.00009	-0.00001	0.0161	0.0276	0.0072
GreatWall	-0.0016	-0.00008	0.0007	0.0145	0.0314	0.0096
House	-0.0004	0.0007	-0.0035	0.0100	0.0278	0.0086
Lake1	0.0018	0.0020	0.0013	0.0147	0.0381	0.0052
Lake2	0.0015	0.0006	0.0021	0.0174	0.0352	0.0068
WhiteTower	-0.0001	0.0041	0.0053	0.0107	0.0276	0.0084
**Average**	**0.0010**	**0.0012**	**0.0023**	**0.0135**	**0.0300**	**0.0072**

Supplementary Table S6| Comparison of correlation coefficients between our algorithm and the quantum image encryption algorithm[44]

Algorithm / Images	Our algorithm			Quantum image encryption algorithm		
	Horizontal correlation	Vertical correlation	Diagonal correlation	Horizontal correlation	Vertical correlation	Diagonal correlation
Buddha	-0.0004	0.0009	0.0006	-0.0005	-0.0006	-0.0025
Flower	0.0015	-0.00009	-0.00001	0.0025	0.0017	0.0032
GreatWall	-0.0016	-0.00008	0.0007	0.0011	0.0004	-0.0049
House	-0.0004	0.0007	-0.0035	-0.0036	0.0099	0.0026
Lake1	0.0018	0.0020	0.0013	-0.0001	0.0055	-0.0018
Lake2	0.0015	0.0006	0.0021	0.0083	0.0009	0.0084
WhiteTower	-0.0001	0.0041	0.0053	0.0009	-0.0052	-0.0029
**Average**	**0.0010**	**0.0012**	**0.0023**	**0.0024**	**0.0035**	**0.0038**

Supplementary Table S7| Comparison of correlation coefficients between our algorithm and the QW-based algorithm[14]

Algorithm / Images	Our algorithm			QW-based algorithm		
	Horizontal correlation	Vertical correlation	Diagonal correlation	Horizontal correlation	Vertical correlation	Diagonal correlation
Buddha	-0.0004	0.0009	0.0006	0.0088	0.0022	0.0020
Flower	0.0015	-0.00009	-0.00001	-0.0022	-0.0074	0.0224
GreatWall	-0.0016	-0.00008	0.0007	0.0064	-0.0131	-0.0072
Lake1	0.0018	0.0020	0.0013	0.0018	0.0070	-0.0179
Lake2	0.0015	0.0006	0.0021	-0.0263	0.0036	0.0075
WhiteTower	-0.0001	0.0041	0.0053	-0.0064	0.0249	0.0029
**Average**	**0.0010**	**0.0012**	**0.0023**	**0.0081**	**0.0098**	**0.0087**

Supplementary Table S8| Comparison of correlation coefficients between our algorithm and the algorithm based on quantum logistic map[45]

algorithm	Proposed algorithm			The quantum algorithm		
	Horizontal	Vertical	Diagonal	Horizontal	Vertical	Diagonal

images	correlation	correlation	correlation	correlation	correlation	correlation
Boat	0.0014	-0.0016	0.0054	0.0065	0.0055	0.0082

Supplementary Table S9| Comparison of correlation coefficients between our algorithm and the algorithm [46]

Algorithm / Images	Our algorithm			Algorithm[46]		
	Horizontal correlation	Vertical correlation	Diagonal correlation	Horizontal correlation	Vertical correlation	Diagonal correlation
Buddha	-0.0004	0.0009	0.0006	-0.0111	-0.0163	0.0061
Flower	0.0015	-0.00009	-0.00001	-0.0004	0.0083	-0.00002
GreatWall	-0.0016	-0.00008	0.0007	0.0060	0.0156	-0.0014
House	-0.0004	0.0007	-0.0035	0.0085	0.0130	0.0119
Lake1	0.0018	0.0020	0.0013	-0.0032	0.0050	-0.0069
Lake2	0.0015	0.0006	0.0021	0.0048	0.0009	0.0066
WhiteTower	-0.0001	0.0041	0.0053	-0.0071	0.0207	0.0064
**Average**	**0.0010**	**0.0012**	**0.0023**	**0.0059**	**0.0114**	**0.0056**

Supplementary Table S10| Comparison of correlation coefficients between our algorithm and the algorithm [47]

Algorithm / Images	Our algorithm			Algorithm[47]		
	Horizontal correlation	Vertical correlation	Diagonal correlation	Horizontal correlation	Vertical correlation	Diagonal correlation
Buddha	-0.0004	0.0009	0.0006	0.0073	-0.0295	-0.0100
Flower	0.0015	-0.00009	-0.00001	0.0018	-0.0689	0.0008
GreatWall	-0.0016	-0.00008	0.0007	0.0067	-0.0645	0.0064
House	-0.0004	0.0007	-0.0035	0.0030	-0.0733	-0.0007
Lake1	0.0018	0.0020	0.0013	-0.0003	-0.0343	0.0093
Lake2	0.0015	0.0006	0.0021	0.0015	-0.0841	0.0053
WhiteTower	-0.0001	0.0041	0.0053	0.0070	-0.0624	0.0076
**Average**	**0.0010**	**0.0012**	**0.0023**	**0.0039**	**0.0596**	**0.0057**

Supplementary Table S11| Results of NPCR and UACI by using our algorithm

Images	NPCR(×100%)	UACI(×100%)
Buddha	99.60	33.50
Flower	99.60	33.48
GreatWall	99.62	33.51
House	99.60	33.44
Lake1	99.60	33.36
Lake2	99.62	33.46
WhiteTower	99.62	33.48

**Average**	**99.61**	**33.46**

Supplementary Table S12| Comparison of NPCR and UACI between our algorithm and the algorithms[41,43]

Algorithm	Average NPCR(×100%)	Average UACI(×100%)
Ours	99.61	33.46
Chaos-based algorithm [41]	0.00004	4.0605e-06
Hash-based algorithm [43]	0.00004	1.4960e-06

Supplementary Table S13| Comparison of NPCR by using our algorithm and the algorithm [46]

Algorithm / Images	Our algorithm	Algorithm[46]
Buddha	99.60	99.18
Flower	99.60	99.17
GreatWall	99.62	99.12
House	99.60	99.01
Lake1	99.60	99.17
Lake2	99.62	99.17
WhiteTower	99.62	99.13
**Average**	**99.61**	**99.14**

Supplementary Table S14| Comparison of UACI by using our algorithm and the algorithm [46]

Algorithm / Images	Our algorithm	Algorithm[46]
Buddha	33.50	23.66
Flower	33.48	24.53
GreatWall	33.51	24.81
House	33.44	22.17
Lake1	33.36	22.96
Lake2	33.46	24.83
WhiteTower	33.48	22.99
**Average**	**33.46**	**23.71**

Supplementary Table S15| Comparison of NPCR between our algorithm and the algorithm [47]

Algorithm / Images	Our algorithm	Algorithm[47]
Buddha	99.60	3.8147e-06

Flower	99.60	0
GreatWall	99.62	0
House	99.60	0
Lake1	99.60	0
Lake2	99.62	0
WhiteTower	99.62	3.8147e-06
**Average**	**99.61**	**0**

Supplementary Table S16| Comparison of UACI between our algorithm and the algorithm [47]

Algorithm / Images	Our algorithm	Algorithm[47]
Buddha	33.50	1.4960e-06
Flower	33.48	0
GreatWall	33.51	0
House	33.44	0
Lake1	33.36	0
Lake2	33.46	0
WhiteTower	33.48	1.4960e-06
**Average**	**33.46**	**0**

Supplementary Table S17| Comparison of NPCR and UACI between our algorithm and the algorithm[45]

algorithm	NPCR(×100%)	UACI(×100%)
Ours	0.996078	33.53
The algorithm[45]	99.5896	33.20

Supplementary Table S18| NPCR value in key sensitivity analysis

Images	NPCR between the encrypted images with different secret keys	NPCR between the decrypted images with correct key and the decrypted images with wrong key
Buddha	99.62	99.58
Flower	99.59	99.61
GreatWall	99.61	99.61
House	99.61	99.61
Lake1	99.61	99.61
Lake2	99.61	99.59
WhiteTower	99.59	99.59
**Average**	**99.61**	**99.60**

Supplementary Table S19| Comparisons of average information entropy of cipher images between our algorithm and other algorithms

Images	Original image	Our algorithm	Chaos-based algorithm	Hash-based algorithm
Buddha	7.357949	7.999175	7.437544	7.997775
Flower	7.445506	7.999297	7.513925	7.994711
GreatWall	7.571477	7.999304	7.852988	7.997933
House	7.466426	7.999226	7.584169	7.998178
Lake1	7.048217	7.999130	7.563206	7.991746
Lake2	7.305189	7.999108	7.785397	7.986923
WhiteTower	7.477779	7.999355	7.692131	7.997278
**Average**	7.381792	**7.999228**	**7.632765**	**7.994934**

Supplementary Table S20| Comparisons of the information entropy of cipher images between our algorithm and the image algorithm based on quantum logistic map[45]

image	Our algorithm	The image algorithm based on quantum logistic map
Boat	7.997	7.999

Supplementary Table S21| Results of NIST SP800-22 for Buddha's cipher image

Test name	P-value	result
Approximate entropy test(block = 10)	0.931323	SUCCESS
Block frequency test(block = 128)	0.566149	SUCCESS
Cumulative sums(forward) test	0.141837	SUCCESS
Cumulative sums (reverse) test	0.091001	SUCCESS
Spectral DFT test	0.102376	SUCCESS
Frequency test	0.846176	SUCCESS
Linear complexity(block = 500)	0.539419	SUCCESS
Longest runs of ones test	0.939260	SUCCESS
Non-Overlapping templates test(m=9,template= 000000011)	0.984629	SUCCESS
Overlapping template of all ones test(m = 9)	0.033341	SUCCESS
Random excursions test(x=-1)	0.766784	SUCCESS
Random excursions variant test(x=+1)	0.857714	SUCCESS
Rank test	0.255072	SUCCESS
Runs test	0.642675	SUCCESS
Serial test1(block = 16)	0.382733	SUCCESS
Serial test2(block = 16)	0.457034	SUCCESS
Universal statistical test(block = 7)	0.135122	SUCCESS
